# Geographical access to care at birth in Ghana: a barrier to safe motherhood

**DOI:** 10.1186/1471-2458-12-991

**Published:** 2012-11-16

**Authors:** Peter W Gething, Fiifi Amoako Johnson, Faustina Frempong-Ainguah, Philomena Nyarko, Angela Baschieri, Patrick Aboagye, Jane Falkingham, Zoe Matthews, Peter M Atkinson

**Affiliations:** 1 Spatial Ecology and Epidemiology Group, Tinbergen Building, Department of Zoology, University of Oxford, South Parks Road, Oxford, United Kingdom; 2 Division of Social Statistics and Demography & Centre for Global Health, Population, Poverty and Policy (GHP3), Faculty of Social and Human Sciences, University of Southampton, Highfield, Southampton, United Kingdom; 3 Regional Institute for Population Studies, University of Ghana, Legon, Ghana; 4 Ghana Statistical Service, Ministry of Finance and Economic Planning (MoFEP), Head Office Building, Accra, Ghana; 5 Ghana Health Services, Accra, Ghana; 6 ESRC Centre for Population Change, University of Southampton, Highfield, Southampton, United Kingdom; 7 Geography and Environment, University of Southampton, Highfield, Southampton, United Kingdom

## Abstract

**Background:**

Appropriate facility-based care at birth is a key determinant of safe motherhood but geographical access remains poor in many high burden regions. Despite its importance, geographical access is rarely audited systematically, preventing integration in national-level maternal health system assessment and planning. In this study, we develop a uniquely detailed set of spatially-linked data and a calibrated geospatial model to undertake a national-scale audit of geographical access to maternity care at birth in Ghana, a high-burden country typical of many in sub-Saharan Africa.

**Methods:**

We assembled detailed spatial data on the population, health facilities, and landscape features influencing journeys. These were used in a geospatial model to estimate journey-time for all women of childbearing age (WoCBA) to their nearest health facility offering differing levels of care at birth, taking into account different transport types and availability. We calibrated the model using data on actual journeys made by women seeking care.

**Results:**

We found that a third of women (34%) in Ghana live beyond the clinically significant two-hour threshold from facilities likely to offer emergency obstetric and neonatal care (EmONC) classed at the ‘partial’ standard or better. Nearly half (45%) live that distance or further from ‘comprehensive’ EmONC facilities, offering life-saving blood transfusion and surgery. In the most remote regions these figures rose to 63% and 81%, respectively. Poor levels of access were found in many regions that meet international targets based on facilities-per-capita ratios.

**Conclusions:**

Detailed data assembly combined with geospatial modelling can provide nation-wide audits of geographical access to care at birth to support systemic maternal health planning, human resource deployment, and strategic targeting. Current international benchmarks of maternal health care provision are inadequate for these purposes because they fail to take account of the location and accessibility of services relative to the women they serve.

## Background

Despite the prominence of international targets for maternal mortality reduction, around a third of a million women continue to die annually from complications of pregnancy or childbirth and many more suffer prolonged or permanent post-partum ill health or disability [1-3]. The greatest share of this burden, around 90%, is borne by developing nations of sub-Saharan Africa and South Asia [2,4]. Limited access to health facilities staffed by appropriately trained personnel and offering midwifery competencies and life-saving obstetric interventions at birth is known to be a key driver of maternal mortality [5,6]. Unlike the vertical programmes of intervention that can be effective in combating other global health challenges [7-9], improving access to effective care at birth relies on strengthening health systems and this partly explains the slow pace of improvement in access and resulting mortality reductions relative to some other global health targets [10,11].

Access to care at birth is determined by a diverse set of factors related to both the services offered (such as their availability, quality and cost) and the population being served (such as their wealth, education, and culturally-mediated perceptions) [10,12,13]. Linking population and health system factors is geography: the physical accessibility of facilities to women in labour. These factors interact in potentially complex ways and in some settings, such as urban areas with robust health infrastructures [14], geographical distance may play only a minor role in determining levels of access and subsequent health outcomes. However, where service provision is sparse, transport infrastructures weak, and populations predominately poor, geography often presents a fundamental and insurmountable barrier to accessing adequate care at birth and therefore plays a central role in sustaining high maternal mortality [15-22]. This is manifest both in those women who die having not sought facility-based care as well as in the significant number that die en route to health facilities, en route from one hospital to another with more appropriate resources, or after arriving too late [20,23,24]. The delays in (i) making the decision to seek care, (ii) reaching an adequate health facility and (iii) receiving the needed care within a facility limits uptake of emergency obstetric care [11,20,25]. While delays (i) and (iii) have received much research attention, delay (ii) has not been studied systematically [26-28].

Given the importance of geographical access, its measurement should arguably form a central component of maternal health system assessment and strategic planning, as well as providing a key development target indicator. Measuring geographical access robustly is, however, fraught with data and methodological challenges and the result is that policy-makers typically revert to crude alternatives such as regional facility-population ratios [29,30]. These ratios are potentially biased and inadequate proxies for auditing the true number of women able to access care at birth and therefore hamper progress towards solutions.

Assessing geographical accessibility over large regions is problematic for several reasons. First, the data requirements are considerable and rarely met in developing countries. Comprehensive data are required on the geographical distribution of both the population and the health facilities to which mothers must travel to access care. Such data sets at the required level of spatial detail, contemporariness, and completeness are the exception rather than rule in SSA [31]. Second, it is well established that straight-line distances act as a poor proxy for the actual cost (distance, time, expense) of journeys [32-35]. This precludes straightforward Euclidean analysis of access and necessitates that the actual landscape across which journeys are made, and the availability of different means of transport, must be known with some degree of detail [36,37]. These factors combine to mean that, whilst the importance of distance to maternal and newborn health services has been demonstrated by many small-scale studies [15-22][38], the detailed measurement of geographical access to maternity care at birth across national populations that might support country-level strategies for scaling up care is rare. Of only two published studies at a national level that are known to the authors, one focuses exclusively on emergency referrals rather than population access to care [39] and the other uses a simple distance measure that is not necessarily representative of actual journeys faced by women in labour [15].

Ghana is representative of most countries in sub-Saharan Africa (SSA) in that rates of maternal mortality remain unacceptably high, with estimates for 2008 of 350 (range of uncertainty 210–630) maternal deaths per 100,000 live births [2]. It is also typical in that (i) a significant proportion of births continue to occur at home without a professionally trained health worker, (ii) the fraction that give birth at home increases dramatically with distance from main urban centres, and (iii) progress in reducing maternal deaths remains slow [6,40]. Geographical access plays a characteristically large role in limiting uptake of maternity care services in Ghana, especially at the time of birth, and was the most commonly cited reason for non-attendance in a recent national survey [6]. Further, a review of 322 maternal deaths occurring in Ghanaian health facilities in 2011 found delay in arrival at a health facility to be a contributing factor in nearly half (46%) of cases. Distance, rather than decision making at home, is strongly implicated in many of these delays [29].

In this study, we use the example of Ghana to present the first detailed national-scale assessment of geographical access to maternity care at birth in a high burden country based on a calibrated journey-time model. This represents one outcome of a four-year study that has included the assembly of comprehensive population, health facility, and landscape data, augmented by multiple existing national and sub-national population sample surveys, and the development of a geospatial framework for modelling realistic journey-times. We use these components to demonstrate the current geographical accessibility of three levels of care at birth in Ghana and identify populations where this is dangerously inadequate. We also compare these results to existing metrics of access, based on regional facilities-per-capita ratios, currently in use by decision-makers.

## Methods

### Overview

**Figure 1:**
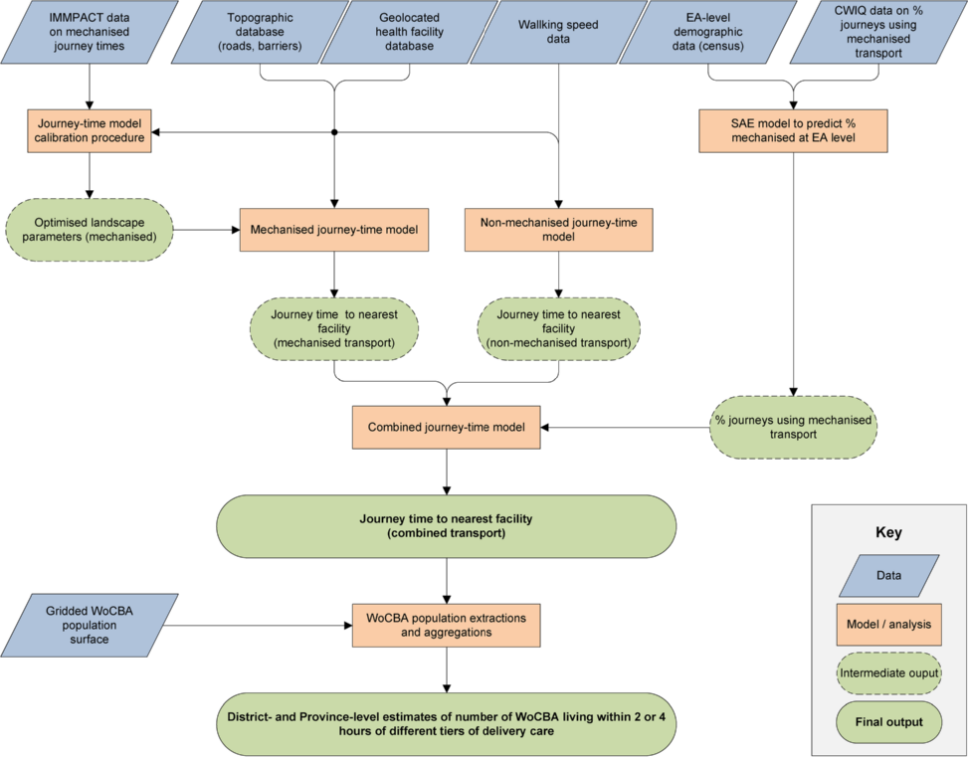
**Schematic diagram showing main input data, analytical steps, and primary outputs in generation of a calibrated nationwide journey-time model.** SAE = small area estimation; EA = enumeration area; WoCBA = women of childbearing age.

The methodological objective of this study was to model geographical access of women to health facilities offering care at birth. Figure 1 shows schematically the various data and modelling components used to achieve this aim. In brief, we assembled a national geo-referenced database of health facilities providing care at birth, and extremely detailed digital topographic data on transport networks (roads, tracks, footpaths) and barriers to travel (e.g. rivers) which were used with a cost-surface algorithm within a geographical information system (GIS) to estimate journey-time from every 100 m × 100 m grid square to the nearest health facility offering a given category of care at birth. Separate models were developed for mechanised versus non-mechanised forms of transport, and the likely proportion of women using each transport type to access care in different locations was estimated using a small-area-estimation (SAE) approach that combined sparse sample survey data with spatially complete census-derived proxies.

To maximise the realism of the model for mechanised journey-times, we carried out an initial calibration stage in which survey data on actual journey-times made by women in labour were used to find optimum model parameters which were then applied nationally. By combining the resulting per-pixel map of journey-times with a high-resolution population map for WoCBA, we generated estimates of the proportion of WoCBA able to access successive levels of care within two hours, within four hours, or more than four hours journey-time. Each of these components is now described in more detailed.

### Establishing a geospatial database of health facilities

We compiled national lists of health facilities from four main sources. First, a list was obtained from the Ghana Ministry of Health containing records of 2,021 facilities of all types nationwide that included for each a description of services offered and the region (first administrative level), district (second administrative level), and town in which each facility was located. Second, a list of geo-referenced facilities was compiled by the Centre for Remote Sensing and Geographic Information Services (CERSGIS), University of Ghana that contained listings of 1,915 facilities nationwide. These two lists were combined, cross-checked and reconciled. The Ghana Ministry of Health also maintains listings of health facilities by district on a web resource. These were cross-checked with the formal listings and any additional facilities added to our database. Facilities without latitude and longitude data were geo-referenced by manual matching of listed town names to mapped locations on Google Earth and, in the remaining unresolved cases, by telephone contact with district health offices to confirm locations. Facilities that do not offer maternity services were excluded (e.g. psychiatric hospitals, supplementary feeding centres, nursing training colleges and administrative offices). Finally, a recent project by the Ghana Ministry of Health and Ghana Health Service carried out an audit of maternity facilities nationwide with accurate assessments of the level of emergency obstetric and neonatal care (EmONC) that each offers. Using an established set of nine 'signal-functions' [30] of potentially life-saving birth care services, all government facilities were classified as non-, partial-, basic- or comprehensive-EmONC depending on the number of signal functions available [29]. All hospitals offering partial-EmONC or higher were extracted from this report, cross-referenced with our existing database, and geo-referenced using the process described above. Where more than one facility was listed at a single site (either because they shared the same building, or because they were geo-referenced using a village location), we retained only the highest order facility for subsequent analysis, thus avoiding any potential duplicated facility listings.

### Grading facilities that provide care at birth

We focused on those health facilities providing care at childbirth, rather than antenatal or postnatal/postpartum care, as this is the crucial period within the continuum of care chain when most mortality occurs, both for women and their newborn babies [5]. We stratified our analysis of geographical access to facilities providing three tiers of care. First, we considered a broad categorisation of 1,864 facilities of all types listed as offering any standard of care at birth (hereafter denoted as any-birth-care facilities, ABC). These spanned the complete spectrum of care from large tertiary hospitals to the most basic peripheral facilities including maternity homes as well as Community-based Health Planning and Services Initiative (CHPS) facilities. Measurement of access to this mixed level of care will overestimate true service availability because many people will be forced to bypass the simpler facilities, many of which offer only rudimentary care and no 24-h staffing. Indeed, even relatively well equipped hospitals in Ghana can offer less than 24-h cover [29]. We included this broad category of health facilities as the theoretical point-of-entry to the health system that also represents a hypothetical best-case scenario were all such facilities fully functional and offering robust referral services.

Second, and representing the other extreme, we considered those 76 hospitals nationwide assessed as offering comprehensive-EmONC services (hereafter C-EmONC). This is a much more stringent designation, denoting hospitals providing all nine signal functions, including the availability of blood transfusion and surgical/caesarean section capability that are typically absent from other facilities. The reality for labouring women in Ghana often lies between these two extremes: a wider set of hospitals offer partial- or basic-EmONC services (where six/seven or eight signal functions are provided, respectively) that nevertheless represent a much higher degree of service than non-EmONC facilities and are able to respond appropriately to a range of birth complications. We therefore assessed access to a third intermediate category that included these partial- and basic- as well as comprehensive-EmONC hospitals (hereafter PBC-EmONC), representing 157 facilities nationwide with heterogeneous levels of care. The final geo-referenced facility database was imported into a GIS (ArcGIS 10.0) as a point shapefile for analysis.

### Establishing a national topographic database

To support geospatial modelling of realistic journey-times to health facilities, detailed topographic datasets were obtained directly from CERSGIS. These included digitised topographic survey data on the national road network and additional tracks and trails, as well as other features such as rivers, lakes, and marshland that may act as a natural barrier to determine the route taken during journeys. These data stem from an unusually detailed national programme of land surveillance carried out by the Water Research Institute, CERSGIS, Department of Feeder Roads, Ghana Survey Department and the Forestry Commission of Ghana between 1995 and 2005. All layers were available as point, polygon, or line feature shapefiles and were imported into ArcGIS for subsequent analysis. These are described in more detailed in Additional file 1.

### Developing a calibrated journey-time model

Cost-surface algorithms are increasingly used within GIS software to estimate journey-times across modelled landscapes [41-47]. Users first define a gridded impedance surface in which the value of each grid cell represents the estimated time required to traverse it, taking into account the size of each cell and the type of landscape feature it represents. Low impedance values are assigned to high-speed features such as roads, with much larger values for off-road or rough terrain. Barrier features can be designated as impassable or assigned very large impedance values. Destination features (e.g. health facilities) are located on the modelled landscape, and the cost-surface algorithm computes the shortest cell-by-cell route from each origin cell to its nearest destination feature. The cumulative sum of impedance values along the route provides the estimated journey-time. The accuracy of these journey-time estimates is dependent on detailed landscape data and on appropriate choice of impedance parameter values.

Cost-surface models have been used to estimate journey-time to health facilities in resource-poor settings, but have often focused only on journeys made on foot [32] or else have dealt simplistically with varying modes of transportation [48]. Pedestrian journeys made without mechanised transport are potentially more straightforward to model because average speeds will tend to be relatively similar across different settings and, because different categories of paths, tracks, and roads offer broadly similar walking speeds, journeys rarely deviate from the most direct route available. We used an established parameter set to represent an average non-mechanised travel speed of 5 kmh^-1^ on established roads or tracks, and 2.5 kmh^-1^ elsewhere [32,49]. Barriers such as rivers and lakes were given higher impedance, meaning journeys were likely to utilise established bridges and crossings where available, but in their absence could be traversed (e.g. by boat), with an appropriate time delay.

Where journeys are made by car, bus, or motorbike, speeds can vary widely according to the type of road or track, and the most direct route is often not the fastest. One approach is to use statutory speed limits as a means of parameterising impedance values for different road categories, but this makes numerous assumptions about road, vehicle, and driver characteristics that may not be valid in many settings. To maximise the realism of our mechanised journey-time model a calibration exercise was undertaken using data on real journeys made by women in labour seeking care in Ghana. Such data have previously been obtained by the IMMPACT [50] project, originally designed to estimate out of pocket costs for birth care [51,52] in which a sample of women giving birth at health facilities in two regions in Ghana (Volta and Central) reported their origin (home) and destination (facility) locations, mode of transport, and time taken to make the journey. From these data, we extracted a total of 138 unique origin–destination pairs and, for each pair, an impedance grid was established from the national topographic data to model the surrounding landscape. These grids differentiated five categories of road or track, from the fastest national highways through to minor paved roads or unmade tracks (see Additional file 1: Figure A1.1). Recognising that the smallest tracks connecting households with the road network may not be captured in the database, a category for 'background' grid cells was defined, within which rivers and lakes were also defined as potential barriers (see Additional file 1: Figure A1.2). An automated algorithm was developed using Python 2.6 which allowed a large number of cost-surface models to be run within ArcGIS 10.0 for each origin–destination journey. In each run, a different set of candidate impedance parameters was assigned to the various landscape features and the resulting estimated journey-times were compared to the values reported in the IMMPACT survey. The overall performance of each parameter set was assessed by the median magnitude of errors between predicted and observed journey-times. A total of 1000 parameter sets were assessed in this way using a hierarchical grid-search that spanned the range of plausible values and the set returning the smallest median absolute error was identified. Optimum impedance parameters were identified as an average travel speed of 60 kmh^-1^ for national roads, 45 kmh^-1^ for inter-regional and regional roads, 5 kmh^-1^ for unmade tracks and trails and 1.75 kmh^-1^ for background pixels.

### Implementation of a nationwide journey-time model

The impedance parameters described above were used to define a mechanised and a non-mechanised impedance grid covering all of Ghana at 100 m × 100 m spatial resolution. Cost-surface algorithms were then implemented for both transport modes to calculate journey-times to nearest ABC, PBC-EmONC and C-EmONC facilities, resulting in a total of six journey-time surfaces. Clearly, journeys made by mechanised transport will almost always be substantially faster than those on foot, and so the availability of transport to women in labour is a critically important determinant of their geographical access to care. This availability will itself be influenced by complex socioeconomic factors that will vary from place to place. To estimate the fraction of women likely to be able to use mechanised transport to seek birth care, we first obtained data collected during the Core Welfare Indicator Questionnaire (CWIQ) survey carried out by the Ghana Statistical Service in 2003 [53]. This is a nationally representative survey which sampled 210,170 individuals from 49,003 households from all the 110 districts^a^ of Ghana and included a question on the mode of transport used in accessing health facilities. This survey was preferred to any direct data on, for example, car ownership, since the journey to seek care is out-of-the-ordinary and may represent a rare occasion when a taxi or bus ride is purchased, or when health facilities themselves may organise transport [6,29]. We used an SAE approach for a unit-level model [54] to relate the CWIQ data to a suite of potential correlates (literacy rate; dwelling ownership; marital status; urban population; material of roof, wall and floor; main source of drinking water; type of toilet facility and main fuel used for cooking) that were also available for all enumeration areas (EAs) from the 2000 Ghana Population and Housing Census [55] and, thereby, impute the fraction of women in each EA likely to make mechanised versus non-mechanised journeys to seek care.

### Assessment of population access to care

As a final step, the six national journey-time maps were combined with a population grid detailing the number of WoCBA (defined as 15–49 years) residing in each 100 m × 100 m grid cell (see Additional file 1: Figure A1.3). This surface is a new product produced by the AfriPop project (http://www.afripop.org) that combines high-resolution census data with satellite sensor imagery of settlements to create the most detailed population surfaces available for Africa [56]. We created policy-relevant summary statistics by summing the number of WoCBA within each district and region that fell within three levels of geographical access: less than two hours from a given facility type, greater than two hours, and greater than four hours. These thresholds follow earlier studies and are based on both clinical factors (two hours being the estimated modal time to death for postpartum haemorrhage [57,58] and empirical analyses showing significant successive increases in maternal case-fatality rates associated with journey-times of greater than two and four hours [59]**.**

### Data access and permissions

All data used in this study were either available on an unrestricted basis in the public domain, or provided with permission from the agencies described above.

## Results

### Modelled journey-time surfaces

**Figure 2:**
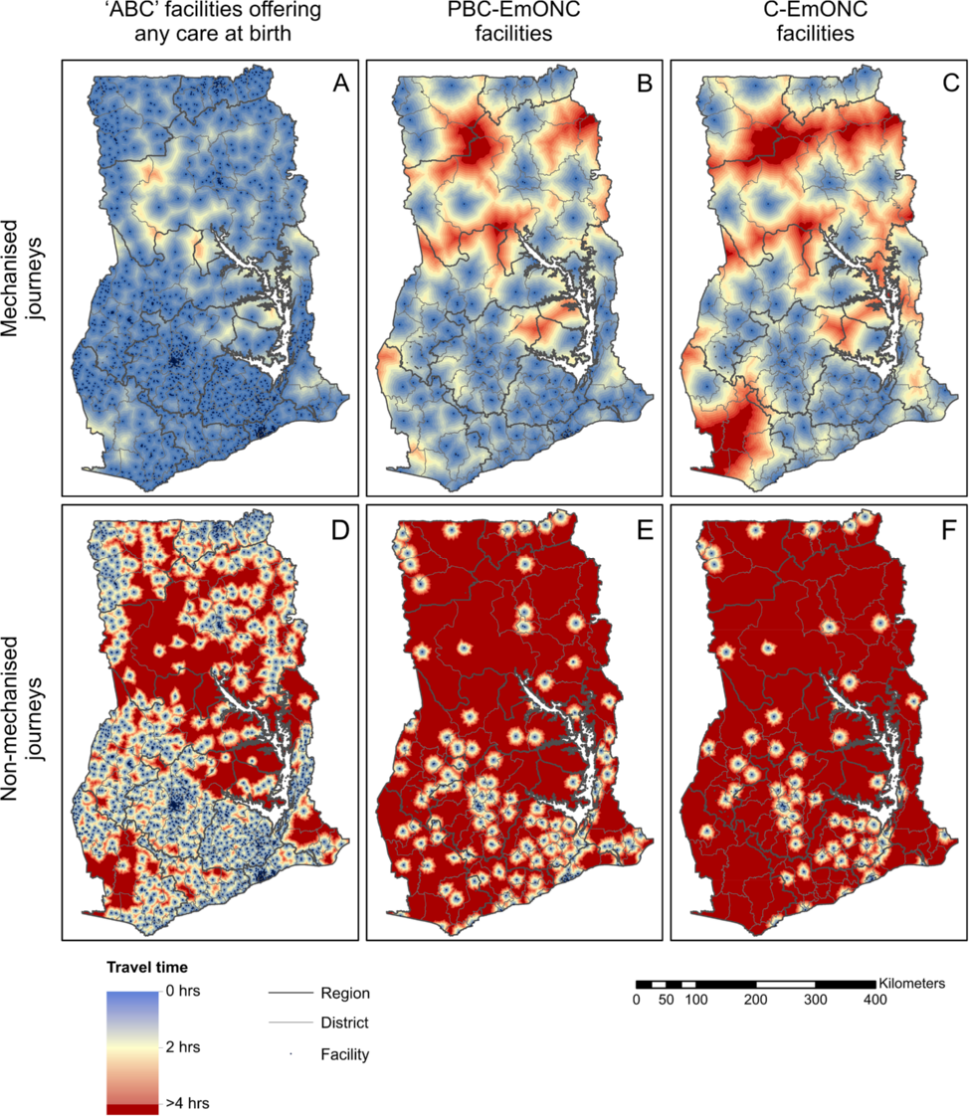
**Modelled journey-time to health facilities offering care at birth.** Mapped values show estimated journey-time via (top row) mechanised and (bottom row) non-mechanised transport from each pixel to the nearest facility offering (**A** and **D**) any level of care at birth; (**B** and **E**) Partial/Basic/Comprehensive EmONC services; or (**C** and **F**) Comprehensive EmONC services. Facilities of each type are overlaid as black dots and district and regional boundaries are also shown.

Figure 2 shows six modelled surfaces estimating for all locations in Ghana the mechanised and non-mechanised journey-time to the nearest ABC, PBC-EmONC and C-EmONC facility. The maps provide a striking visual indication of the degree of variation in levels of geographical access, both between different areas of the country and between the different grades of facility for care at birth. When considering access to the broadest category of care offered by the 1,864 ABC facilities nationwide, most parts of the country lay within a two-hour mechanised journey (Figure 2A), as indicated by the predominantly blue shading, although drive-times exceeded this threshold in the most remote rural areas (e.g., in Northern region). Geographical access to higher standards of facility care, however, was substantially poorer for much of the country. Even with the use of mechanised transport, significant proportions of the northern half of Ghana lay more than two hours journey-time away from PBC-EmONC facilities (Figure 2B), as indicated by the yellow and red shades. The same areas, along with a large part of Western region in the south-west, were often more than four hours mechanised journey-time from C-EmONC (Figure 2C) facilities. Unsurprisingly, predicted levels of access were dramatically poorer when accessed via non-mechanised transport. Even when considering access to the widespread ABC facilities, much of the country lay more than two hours journey-time away (Figure 2D), and for PBC-EmONC (Figure 2E) and C-EmONC (Figure 2F) facilities, the overwhelming majority of the country lay beyond four hours journey-time.

### Geographical accessibility at the national level

When the various journey-time models displayed in Figure 2 were combined, and taking into account the predicted use of mechanised versus non-mechanised transport, we estimated that 90% of WoCBA in Ghana were within two hours journey-time of their nearest ABC facility, 66% within two hours of their nearest PBC-EmONC facility, and 55% within two hours of their nearest C-EmONC facility (Table 1). At the national level, therefore, whilst most of the at-risk population can reach some form of facility offering birth care within a clinically appropriate timeframe, a third lie beyond this safe journey- time to hospitals able to offer at least partial-EmONC services, and this proportion rises to nearly a half when the more stringent comprehensive-EmONC criteria are applied. Some 29% of WoCBA reside more than four hours from this latter class of facility, representing nearly 1.8 million women who are unlikely to be able to reach life-saving blood transfusion or surgery in time to avert death from post-partum haemorrhage.

**Table 1:** Summary of geographical access to three levels of facility-based birth care in Ghana, by region

**Region**	**Travel time category**	**‘ABC’ facilities offering any care at birth**	**EmONC facilities (partial, basic or comprehensive)**	**EmONC facilities (comprehensive)**
**NATIONAL***Total = 6,205,703*	< 2 hrs	5,586,265 (90%)	4,099,329 (66%)	3,384,040 (55%)
	> 2 hrs	619,438 (10%)	2,106,374 (34%)	2,821,663 (46%)
	> 4 hrs	185,917 (3%)	1,091,760 (18%)	1,766,669 (29%)
**Ashanti***Total = 1,796,118*	< 2 hrs	1,708,397 (95%)	1,315,565 (73%)	1,223,834 (68%)
	> 2 hrs	87,721 (5%)	480,553 (27%)	572,284 (32%)
	> 4 hrs	12,561 (1%)	197,955 (11%)	276,998 (15%)
**Brong Ahafo***Total = 548,345*	< 2 hrs	479,302 (87%)	318,954 (58%)	269,541 (49%)
	> 2 hrs	69,043 (13%)	229,391 (42%)	278,803 (51%)
	> 4 hrs	11,851 (2%)	133,026 (24%)	189,944 (35%)
**Central***Total = 496,948*	< 2 hrs	430,841 (87%)	297,882 (60%)	250,054 (50%)
	> 2 hrs	66,107 (13%)	199,066 (40%)	246,894 (50%)
	> 4 hrs	7,534 (2%)	106,144 (21%)	195,614 (39%)
**Eastern***Total = 624,211*	< 2 hrs	600,380 (96%)	426,226 (68%)	394,962 (63%)
	> 2 hrs	23,831 (4%)	197,986 (32%)	229,249 (37%)
	> 4 hrs	8,000 (1%)	64,969 (10%)	110,305 (18%)
**Greater Accra***Total = 785,183*	< 2 hrs	782,777 (99%)	736,523 (94%)	644,211 (82%)
	> 2 hrs	2,406 (1%)	48,660 (6%)	140,972 (18%)
	> 4 hrs	0 (0%)	19,734 (3%)	63,860 (8%)
**Northern***Total = 551,093*	< 2 hrs	428,607 (78%)	205,709 (37%)	139,996 (25%)
	> 2 hrs	122,486 (22%)	345,383 (63%)	411,096 (75%)
	> 4 hrs	37,463 (7%)	228,182 (41%)	276,431 (50%)
**Upper East***Total = 551,093*	< 2 hrs	230,331 (98%)	130,994 (56%)	122,261 (52%)
	> 2 hrs	4,245 (2%)	103,582 (44%)	112,314 (48%)
	> 4 hrs	43 (0%)	45,690 (20%)	82,763 (35%)
**Upper West***Total = 160,357*	< 2 hrs	145,442 (91%)	94,318 (59%)	56,933 (36%)
	> 2 hrs	14,915 (9%)	66,039 (41%)	103,423 (65%)
	> 4 hrs	1,735 (1%)	38,352 (24%)	73,455 (46%)
**Volta***Total = 472,916*	< 2 hrs	340,811 (72%)	273,282 (58%)	178,094 (38%)
	> 2 hrs	132,105 (28%)	199,634 (42%)	294,822 (62%)
	> 4 hrs	65,228 (14%)	114,732 (24%)	209,138 (44%)
**Western***Total = 535,956*	< 2 hrs	439,376 (82%)	299,875 (56%)	104,152 (19%)
	> 2 hrs	96,580 (18%)	236,081 (44%)	431,804 (81%)
	> 4 hrs	41,502 (8%)	142,975 (27%)	288,162 (54%)

Values are the estimated number (and regional percentage) of women of childbearing age living less than 2 hours, greater than 2 hours or greater than 4 hours journey-time from their nearest facility offering each level of care. Also shown in the left-hand column are the estimated total number of women of childbearing age in each region. EmONC = emergency obstetric and newborn care.

### Geographical accessibility at the regional and district level

**Figure 3:**
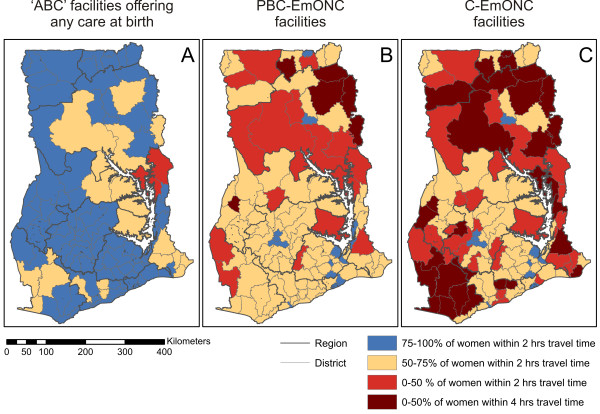
**District level summaries of estimated geographical access by women of childbearing age to nearest health facilities offering care at birth.** Panels relate to facilities offering (**A**) any level of care at birth; (**B**) Partial/Basic/Comprehensive EmONC services; and (**C**) Comprehensive EmONC services. District and regional boundaries are also shown.

The national-level summary estimates described above obscure marked variation in levels of geographic access across Ghana. Table 1 provides estimates disaggregated to the country's ten regions. The highest levels of geographical access are found in the metropolitan region of Greater Accra, which is almost exclusively urban and incorporates the national capital. There, 82% of WoCBA can access C-EmONC facilities within two hours, with only 8% beyond four hours. In contrast, the two predominantly rural Northern and Western regions have a quarter or less of WoCBA with access to C-EmONC within two hours (25% and 19% respectively) and in both cases at least half (50% and 54%) are beyond four hours from those facilities. Figure 3 provides equivalent estimates at the district level. Within-region variation is pronounced in most of the ten regions, especially for access to EmONC facilities (Figure 3B and C). In total 29 (of 110) districts fell into the worst access category with less than 50% of WoCBA living within four hours of their nearest C-EmONC facility.

### Comparison with international metrics of access

The World Health Organization has set an international target for provision of one C-EmONC facility per 500,000 population [29] and this has been extended by the Government of Ghana to a more stringent target of one per 200,000 [30]. Targets defined with these ratio-based metrics are easy to assess, but may not adequately represent the complexity of varying geographical access within a given administrative region. In Table 2, we list the availability of C-EmONC facilities in each of the ten Ghanaian regions with respect to the national and international targets, and compare these metrics with our own assessment of geographical access to the same facilities. The inadequacy of the international 1:500,000 ratio is immediately apparent. Seven of the ten regions exceed this target, and yet in those same regions large proportions of women are unable to access C-EmONC facilities within safe journey-times. In Upper West region, for example, there is approximately one C-EmONC facility per 170,000 population, thus exceeding the international target by a factor of three. We estimate, however, that only 35% of WoCBA in that region can access these facilities within two hours, and only 54% within four hours. In Northern region, which provides one C-EmONC facility per 300,000 population, we estimate that only 25% and 50% can access a C-EmONC facility within two and four hours, respectively.

**Table 2:** Comparison of national and international targets for provision of comprehensive EmONC facilities with estimated levels of geographic access

**Region**	**Population per C-EmONC facility**^**1**^	**Provision of C-EmONC facilities: % of target achieved**^**2**^		**% women with geographical access to C-EmONC facilities**	
		**UN target (1:500,000)**	**National target (1:200,000)**	**within 2hrs**	**within 4hrs**
**NATIONAL**	318,848	**157%**	63%	54%	71%
**Ashanti**	262,503	**190%**	76%	68%	85%
**Brong Ahafo**	228,213	**219%**	88%	49%	65%
**Central**	526,802	**95%**	38%	50%	61%
**Eastern**	185,430	**270%**	**108%**	63%	82%
**Greater Accra**	434,418	**115%**	46%	82%	92%
**Northern**	308,570	**162%**	65%	25%	50%
**Upper East**	343,826	**145%**	58%	52%	65%
**Upper West**	169,441	**295%**	**118%**	35%	54%
**Volta**	699,959	71%	29%	38%	56%
**Western**	775,199	64%	26%	19%	46%

Percentages exceeding stated targets are highlighted in bold.

1. Population ratios derived from [29] with population data based on provisional results from 2010 Ghana Statistical Service Population and Housing Census. 2. Targets as stated in [29] and [30].

## Discussion

Efforts to reduce maternal mortality by improving the quality and availability of facility-based care during childbirth will have limited impact where long distances, poor infrastructure, and lack of transport mean women are unable to physically access these services within clinically appropriate timeframes. Because geographical access is difficult to measure across entire populations, the international community has defined targets based on facility provision per capita. Here, we have used a uniquely detailed assembly of spatial data on the population, health service and topographic landscape, as well as supporting survey and census data to reconstruct realistic levels of geographical access to life-saving delivery care at a national scale. This has allowed us to audit the number of women in Ghana with dangerously poor levels of access, but also to assess the appropriateness of international targets as a benchmark indicator of adequate service provision.

We have found that geographical access in Ghana is generally poor with long distances to health facilities particularly in the rural areas. Although a high proportion (90%) of women in Ghana have relatively good access to some form of facility offering care at birth, this must be interpreted in the context of low levels of service provision at the majority of these facilities. A substantial proportion of women (around a third) live beyond two hours from any facility likely to offer partial EmONC or above and nearly half live that distance or further from comprehensive EmONC facilities offering life-saving blood transfusion and surgery. Worryingly, nearly a third of women live more than four hours from these top-tier facilities and are, thus, at substantially greater risk of dying in the event of unforeseen complications during childbirth. Our analysis also highlights the marked regional variation in geographical access, and allows detailed identification of the worst-served communities. By comparing our results to international and national targets assessed sub-nationally, we also highlighted how crude per-capita facility provision ratios can mask the actual levels of access for populations living distant from urban centres. This has potentially important consequences that extend beyond the Ghanaian context: in any setting with dispersed rural populations, the meeting of international targets is unlikely to be a guarantee of safe accessibility for large proportions of women.

### Recommendations for action

A more positive interpretation of our results is that, taking into account the predicted use of mechanised versus non-mechanised transport, 90% of WoCBA in Ghana are within two hours journey-time of their nearest birthing facility of some kind. Although most of these facilities do not meet formal standards of care, many do have the capacity - or the potential, if strengthened - to deal with some complications and refer women if necessary. The Millennium Development Goals Acceleration Framework (MAF, [60]), as planned for 2012–2015 by the Ghana Health Services with the support of a large range of stakeholders has the potential to strengthen those facilities that currently lack the capability to deal with such complications, as well as to build up capacity for referral. The results presented in this study can support the optimum targeting of this initiative to maximise improvements in geographical access to underserved populations. Weak referral infrastructure is a key bottleneck identified by the MAF and actions should be designed to strengthen transport solutions such as ambulances and taxi drivers unions, as well as communications technologies. These initiatives must be set against the context of a growing demographic challenge in Ghana, as the number of births continues to grow due to the persistence of high fertility rates [40].

### Limitations, caveats, and generalisability

We chose to focus in this study on access to facility-based care at birth. Whilst provision of services for safer home-based birth can form an important part of care provision in some settings, a facility-centred intrapartum-care strategy is increasingly advocated [11] and reflects the fact that an estimated 10-15% of women will develop unexpected and potentially life-threatening complications requiring emergency obstetric care [4,30,61]. We also do not consider in this analysis the potential role of antenatal care in identifying potentially high risk deliveries, but recognise that this may form an important part of an improved access strategy.

Modelling journeys accurately is a particularly complex challenge. A huge variety of factors and decision-making processes contribute to the circumstances of each individual journey made to seek care. Although we have compiled an unusually comprehensive set of data describing the landscape, it is also true that it will never be possible to capture local details that may in some cases be important. One simplification we have made is to assume a static landscape. Clearly, the quality of unmade road surfaces can vary dramatically between wet and dry seasons, and this is not captured directly in our model. The survey data on actual journey-times used to calibrate the model were, however, obtained during a 12-month period and therefore captured a spread of journey-times across all seasons [62]. We also make some simplifying assumptions regarding travel speed and traffic flows. In urban areas and, in particular, the large conurbations of Accra and Kumasi, journey-times are often determined not by the quality of roads, but by heavy traffic which can make even short journeys extremely slow. Our calibration data were from predominately rural areas and so these effects were not captured in our model.

The analytical framework we have presented in this study can, in principle, be envisaged in any country as part of an evidence-based approach to assessing geographical access to care at birth or indeed to other facility-based health services. The extent of generalisability to new settings is likely to be determined almost entirely by the availability of adequate data, including detailed geospatial data on landscape features and facility service audits linked to georeferenced facility locations. The increasing availability of such data sources bodes well for the application of similar work across a broader swathe of countries.

## Conclusion

The journey to an adequate health facility can represent an insurmountable barrier to women during childbirth. As the international community seeks to accelerate efforts to reduce maternal mortality, the measurement of this barrier becomes increasingly important as a platform for evidence-based strategies to improve access to care. We show here that detailed country-wide measurement of geographical access to care can be achieved in a high burden country, and that such analysis has revealed for Ghana the nature of accessibility to their evolving network of facilities. We also demonstrate how a GIS study of this type can significantly enhance the systemic information available to plan services, deploy human resources, and provide strategic intelligence to a range of policymaking efforts including the effective targeting of poorly accessible services for strengthening. Clearly, international benchmarks of service provision are inadequate for these purposes.

## Endnotes

^a^The districts in this study refer to the 110 districts created as part of the political decentralisation of Ghana in 1988 and adopted for the 2000 Ghana Population and Housing Census. Additional districts have been created recently.

## Abbreviations

ABC: Any-birth-care’ facilities; CERSGIS: Centre for Remote Sensing and Geographic Information Services; CHPS: Community-based Health Planning and Services Initiative; CWIQ: Core Welfare Indicator Questionnaire; EA: Enumeration areas; EmONC: Emergency obstetric and neonatal care; C-EmONC: Comprehensive-EmONC; PBC-EmONC: Partial- basic- or comprehensive-EmONC; GIS: Geographical information system; MAF: Millennium-Development-Goals Acceleration Framework; SAE: Small-area-estimation; SSA: Sub-Saharan Africa; WoCBA: Women of childbearing age.

## Competing interests

The authors declare that they have no competing interests.

## Authors’ contributions

PWG, PMA, and ZM conceived the analyses and PWG wrote the first draft of the manuscript. PWG, PMA and FAJ led development of the modelling architecture. FAJ, FFA, PN, PWG and ZM assembled and processed the databases. PN, AB, PA, JF contributed additional data, analyses, and writing. All authors contributed to refining the experiments and the final version of the manuscript.

## Pre-publication history

The pre-publication history for this paper can be accessed here:

http://www.biomedcentral.com/1471-2458/12/991/prepub

## Supplementary Material

Additional file 1 Establishing a National Topographic Database. Click here for file
