# Supplementary material for: Geographical access to care at birth in Ghana: a barrier to safe motherhood
Source: BMC Public Health. 2012 Nov 16;12:991. doi: 10.1186/1471-2458-12-991 (PMC3533981; doi:10.1186/1471-2458-12-991)
Supplement: Additional file 1 — Establishing a National Topographic Database. [file 1471-2458-12-991-S1.docx]

**Additional file 1: Establishing a National Topographic Database**

This document displays and provides additional information on the national geospatial and topographic data that were assembled from numerous sources, cleaned and reconciled to support implementation of the journey-time modelling at the national scale.

A1.1 Transport Features

Figure A1.1 displays the digitised road network data that underpinned our model for mechanised transport times. These data were obtained from the Centre for Remote Sensing and Geographic Information Services (CERSGIS) [[1](#_ENREF_1)], University of Ghana, and were originally generated by a surveillance project led by the Department of Feeder Roads. All engineered and non-engineered roads were surveyed and digitised using Global Positioning Systems (GPS) technology, with data archived in ArcInfo geographic information system (GIS). Roads were classified as national routes (the trunk road system in Ghana connecting the major regional cities); inter-regional and regional routes (the second-tier roads that complete the national network); with non-engineered surfaces classified as tracks or trails.

A1.2 Hydrological Features

Figure A1.2 displays the digital survey data on river and stream channels and permanent water bodies in Ghana. These were incorporated as potential impediments to travel, with journeys (particularly those on foot) routed to circumvent these features in the absence of a road crossing. These data were again obtained from CERGIS, and originate from a national programme of hydrological feature surveillance undertaken by the Water Research Institute and Ghana Survey Department.

A1.3 Population Data

Figure A1.3 displays the gridded population data that were used to determine the spatial distribution of women of childbearing age (WoCBA) with respect to the location of health facilities offering maternal health services. This surface shows estimates of the total number of women aged 15-49 years living in each 100m × 100m grid cell. These data originated from the AfriPop project ([www.afripop.org](http://www.afripop.org)) which uses a standardised approach to assemble contemporary census data and link these to ancillary remote sensing and gazetteer data on settlement locations and sizes to generate the finest resolution population data available for Africa [[2](#_ENREF_2)].

A1.4 Administrative Region Data

Figure A1.4 displays the digitised boundary data delineating the administrative regions of Ghana. These relate to the regions as defined at the time of the 2000 Population and Housing Census [[3](#_ENREF_3)] and we present three tiers of geographic units: regions (1^st^ level), districts (2^nd^ level), and enumeration areas (census enumeration unit). These data were obtained directly from the Ghana Statistical Service and were used to link census variables to national survey data from the CWIQ survey as part of the small-area estimation model to predict access to mechanised transport.

**References**

1. Mensa M, Sablah E, Amamoo-Otchere E, Mensah F (2006) Digital Mapping and GIS-Driven Feeder Road Network Database Management System for Road Project: Planning and Implementation Monitoring in the Feeder Road Sector. Accra, Ghana: Centre for Remote Sensing and Geographic Information Services (CERSGIS).

2. Linard C, Gilbert M, Snow RW, Noor AM, Tatem AJ (2012) Population distribution, settlement patterns and accessibility across Africa in 2010. PLoS One 7: e31743.

3. Ghana Statistical Service (2002) 2000 Population and Housing Census: Summary Report of Final Results. Accra: Ghana Statistical Service.


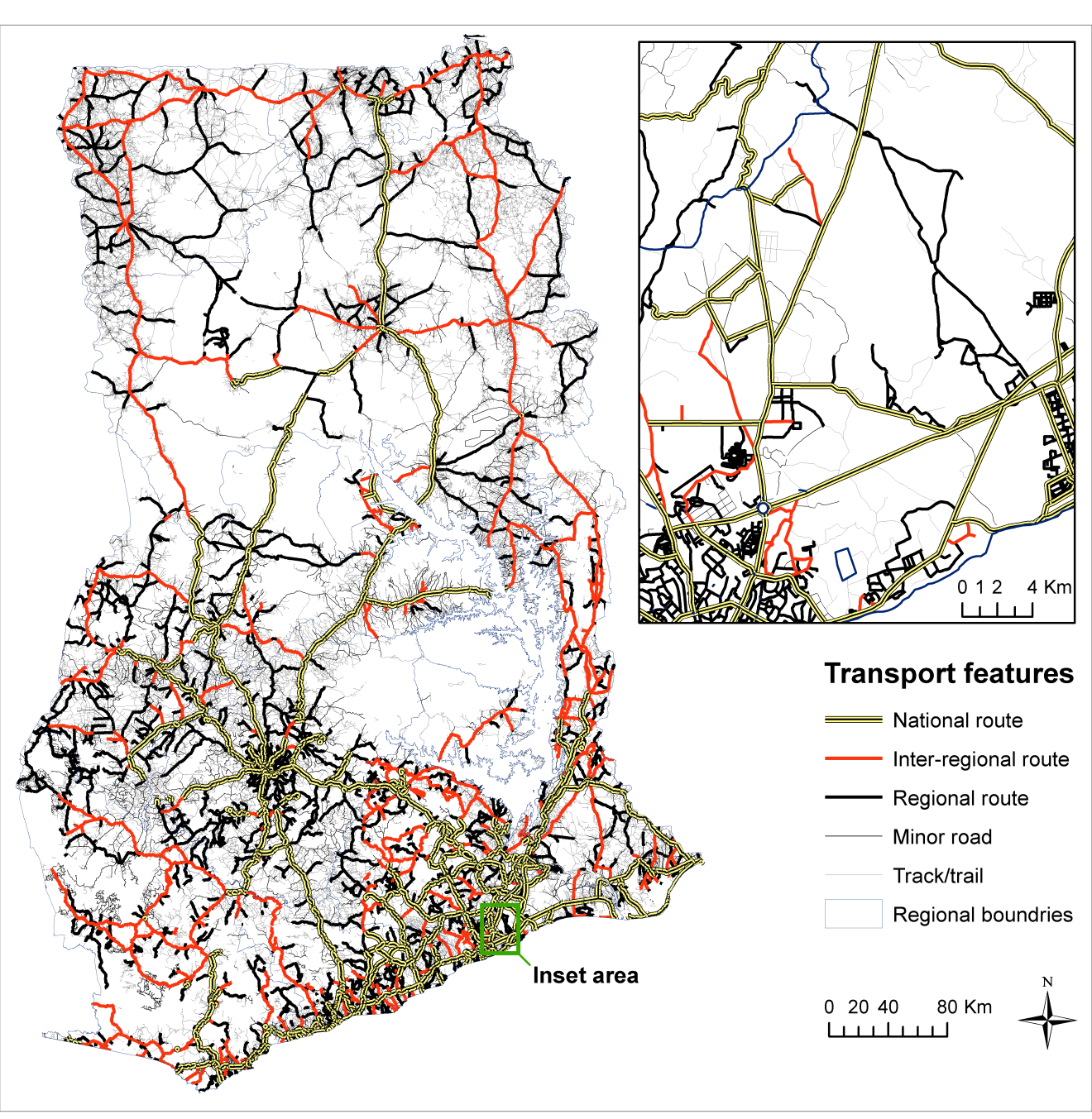


**Figure A1.1. Digital survey data on the Ghana road network.** Data were available as an ArcGIS vector shapefile and defined five hierarchical classes of road, from major National highways to unmade tracks and trails. An inset area is provided to illustrate the level of local detail in these data.


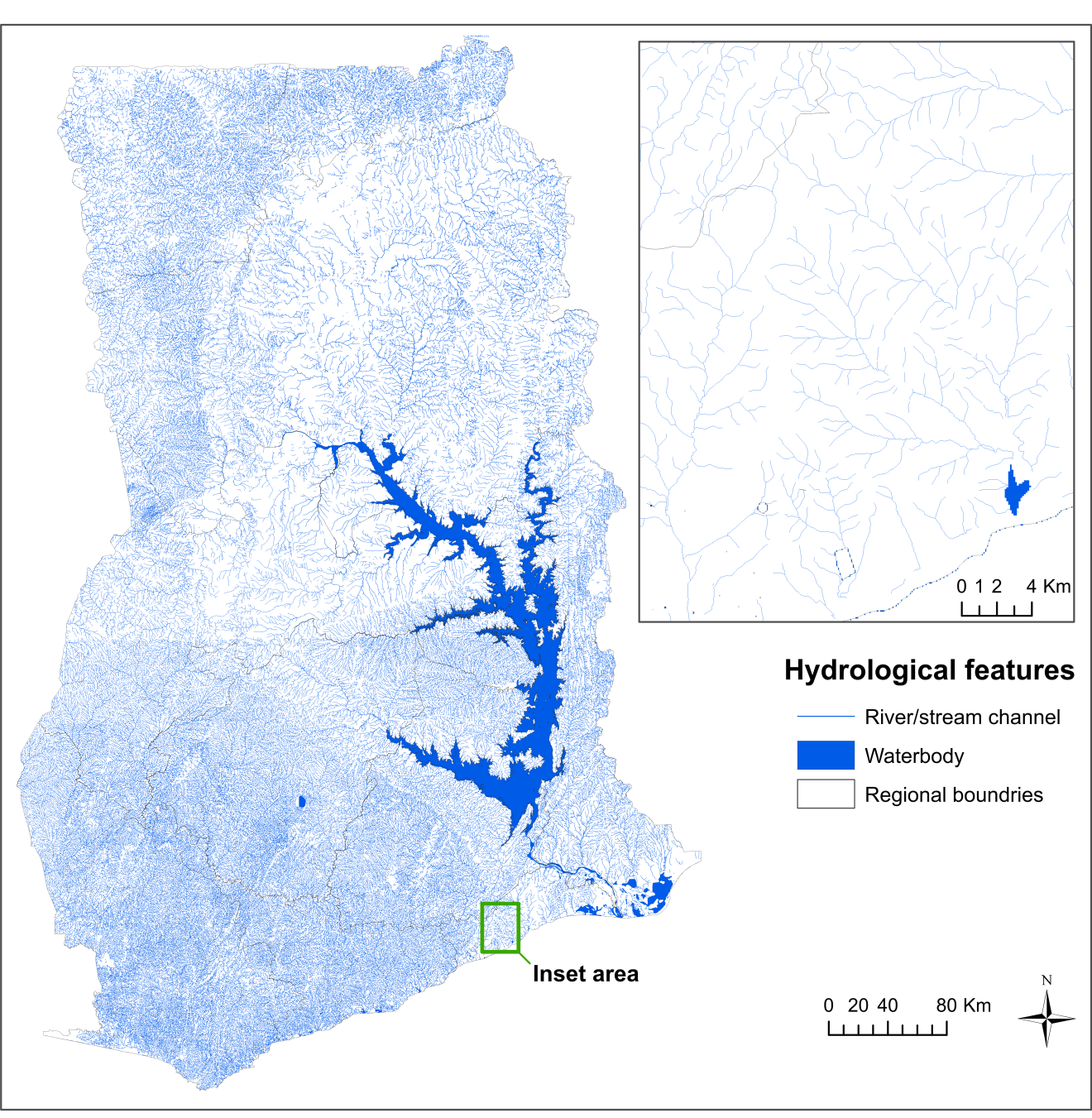


**Figure A1.2 Digital survey data on hydrological features in Ghana.** Data were available as an ArcGIS vector shapefile for both river and stream channels and permanent waterbodies (ponds, lakes, reservoirs). An inset area is provided to illustrate the level of local detail in these data.

**
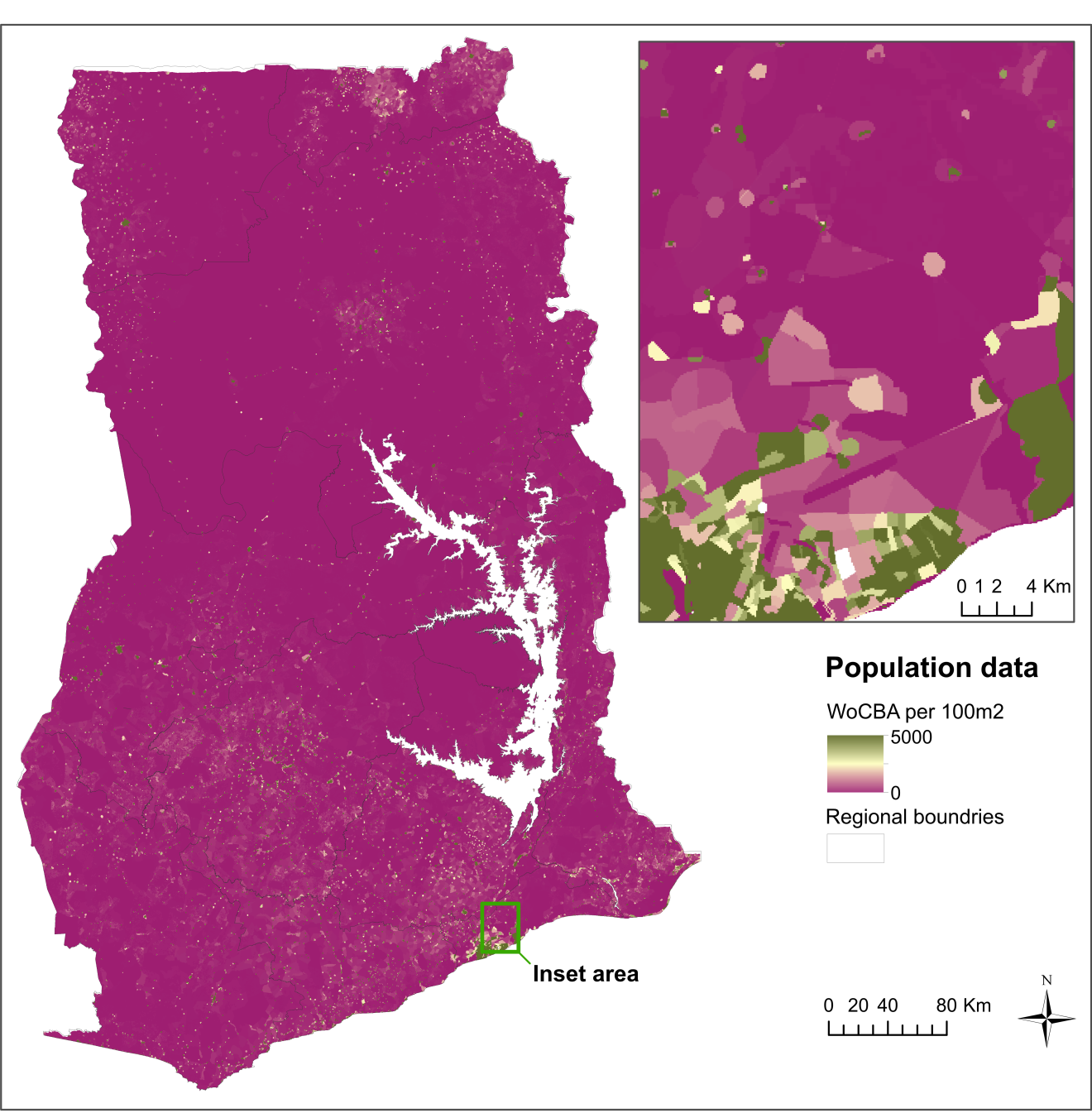
**

**Figure A1.3 Digital population surface.** An ArcGIS raster grid surface at 100m ×100m resolution showing the estimated number of women of childbearing age (WoCBA, defined as 15-49 years) within each grid cell. An inset area is provided to illustrate the level of local detail.

**
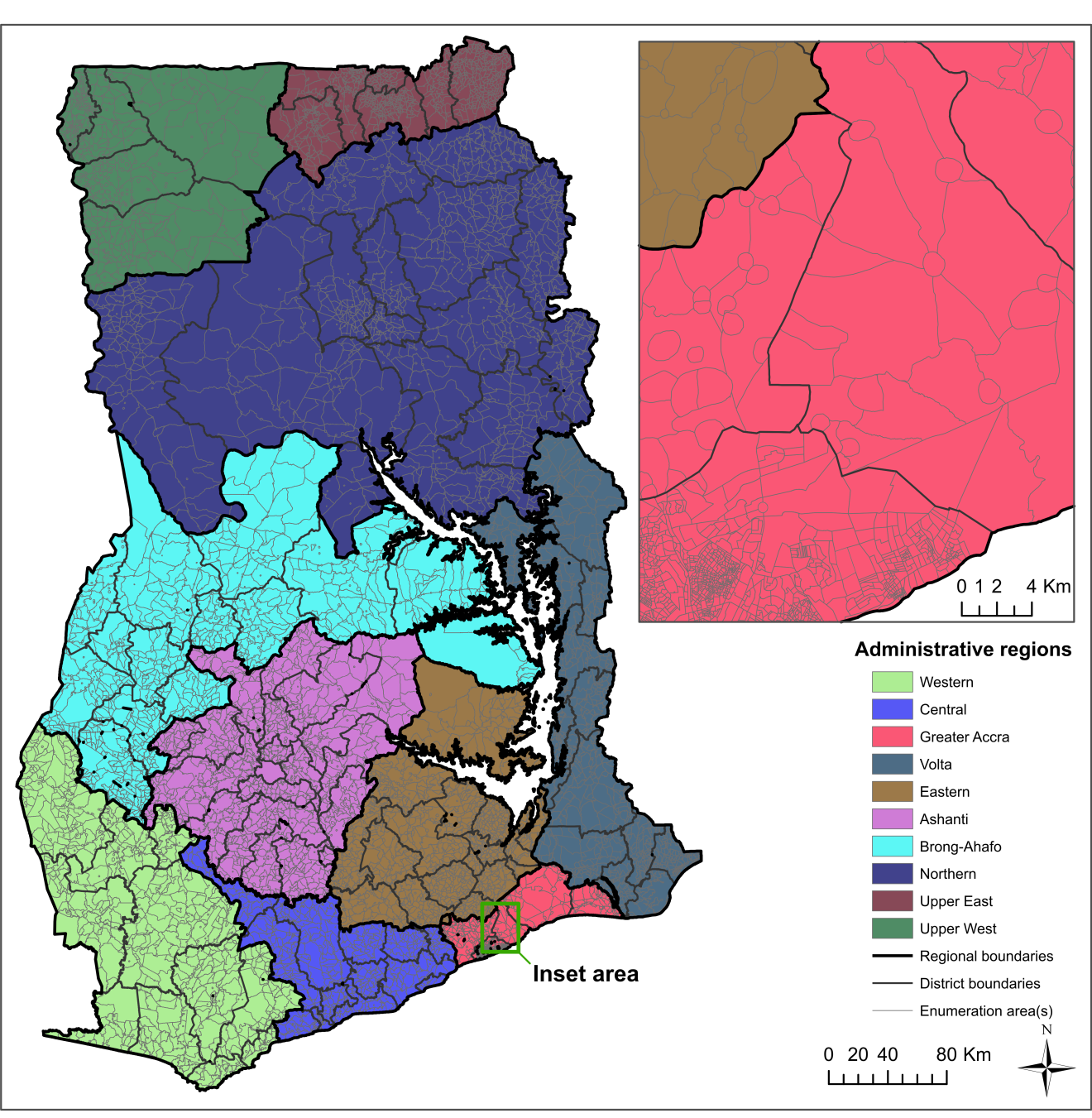
**

**Figure A1.4 Digitised administrative boundary data.** Data were available as an ArcGIS vector shapefile obtained directly from the Ghana Statistical Service and relate to divisions defined for the 2000 Population and Housing Census. Regions, districts, and enumerations areas are shown, with each region coloured separately. An inset area is provided to illustrate the level of local detail in these data.
